# The Role of HIF-1α in Atrial Fibrillation: Recent Advances and Therapeutic Potentials

**DOI:** 10.31083/RCM26787

**Published:** 2025-02-20

**Authors:** Feng Zhou, Jia-Bin Zhou, Tian-Peng Wei, Dan Wu, Ru-Xing Wang

**Affiliations:** ^1^Department of Cardiology, The Affiliated Wuxi People’s Hospital of Nanjing Medical University, Wuxi People’s Hospital, Wuxi Medical Center, Nanjing Medical University, 214023 Wuxi, Jiangsu, China

**Keywords:** hypoxia-inducible factor 1-alpha, atrial fibrillation, mechanism

## Abstract

The steady increase in life expectancy throughout the world is contributing to an increased incidence of atrial fibrillation (AF), which imposes a significant socioeconomic toll on affected patients and societies. The mechanisms underlying atrial fibrillation are multifaceted and vary among individuals. Hypoxia is a process that is closely linked to AF onset and progression. Hypoxia-inducible factor 1-alpha (HIF-1α) is a transcription factor that serves as a key regulator of oxygen homeostasis within cells through its activation under hypoxic conditions and subsequently coordinates various pathophysiological responses. High levels of HIF-1α expression are evident in AF patients, and facilitate the progression from persistent AF to permanent AF. Thus, HIF-1α may serve as a promising target for novel therapeutic strategies aimed at the prevention and treatment of AF. This review provides an overview and synthesis of recent studies probing the relationship between HIF-1α and AF, providing a foundation for future studies and the development targeted drug therapies.

## 1. Introduction

Atrial fibrillation (AF) is a commonly diagnosed form of sustained arrhythmia, affecting millions of patients worldwide and increasing their risk of both heart failure and stroke. The complex etiological basis for AF has been characterized in great detail in recent years, highlighting roles for structural remodeling, electrical remodeling, calcium ion handling abnormalities, and dysregulated autonomic nervous system activity [1]. Furthermore, AF is closely linked to hypoxia. Meanwhile, hypoxia-inducible factor (HIF)-1α functions as a central coordinator of oxygen homeostasis within cells, and its expression in both cardiac myofibroblasts and cardiomyocytes is thought to be relevant to AF development [2]. This review provides a detailed overview of the functional role of HIF-1α in AF and discusses its potential therapeutic implications, thereby laying a foundational knowledge base to support future therapeutic strategies against this disruptive form of arrhythmia.

## 2. The Pathophysiology of Atrial Fibrillation

AF is characterized by an irregular, rapid atrial rhythm that is linked to a higher risk of heart failure, stroke, dementia, cognitive dysfunction, and mortality [3]. These affected patients experience a reduction to overall quality of life [3]. With the accelerating aging of the global population, AF is forecast to impose an increasingly heaving burden on healthcare systems and societies in the coming decades.

Electrical and structural remodeling, together with the dysfunction of the autonomic nervous system and calcium homeostasis, are closely tied to AF progression [4]. AF is characterized by the abnormal expression of many ion channels, including L-type Ca^2+^ channels (ICa,L), late Na^+^ channels (INa,L), and voltage-gated K^+^ channels (Kv) [5]. These changes contribute to a reduction in the atrial effective refractory period (ERP) and action potential duration (APD), contributing to emergent conduction disturbances that result in a feedforward loop that fuels further AF progression through persistent electrical remodeling [5].

Myocardial fibrosis plays a central role in atrial remodeling in affected patients. Specifically, the formation of fibrotic tissue can physically separate longitudinal atrial myofibers such that muscle discontinuities form, establishing a physical barrier to local signal conduction in the atrium. Interactions between fibroblasts and cardiomyocytes can also alter cardiomyocyte conduction properties, in turn triggering ectopic discharges and AF [6]. Both cardiac sympathetic and parasympathetic nervous system activity are involved in the development of AF. AF, in turn, can alter levels of autonomic excitability, consistent with the mutually reinforcing effects of autonomic dysfunction and AF [7]. The loss of appropriate intracellular Ca^2+^ homeostasis is also linked to AF incidence, with abnormal Ca^2+^ channel density, for instance, leading to atrial APD shortening and elevated Ca^2+^ levels within cells. These ions can then activate protein kinase C (PKC), engaging a downstream signaling axis that culminates in further structural remodeling and AF onset [3].

## 3. The Structural and Functional Properties of HIF-1α

Members of the HIF family of hypoxia-sensitive transcription factors are central to the ability of cells to detect oxygen availability and to regulate oxygen homeostasis [8]. HIF-1 is a member of this family that is composed of the HIF-1α and HIF-1β subunits, which are universally expressed across mammalian cells, with HIF-1α serving as the primary regulator of HIF-1 activity [9].

The *HIF1A *gene encodes HIF-1α, which is expressed at high levels in tissues exposed to hypoxic conditions and can be rapidly activated by exposure to severe acute hypoxia (1–2% O_2_) [2]. Under these conditions, it controls anaerobic glycolytic activity or cell death [2]. Under conditions with normal O_2_ levels, the hydroxylation of HIF-1α by prolyl-4-hydroxylase (PHD) enzymes, leads to its proteasomal degradation through a process mediated by von Hippel-Lindau protein (pVHL), an E3 ubiquitin ligase [10]. The HIF-1α Asn-803 residue can also be hydroxylated by factor inhibiting HIF (FIH) [11], interfering with its ability to bind to transcriptional co-activators including CREB-binding protein (CBP)/p300 [12].

Exposure to hypoxia leads to the disruption of PHD and FIH activity, preventing HIF-1α from undergoing the associated post-translational modifications. It instead translocates to the nucleus and heterodimerizes with HIF-1β, after which the HIF-1α-HIF-1β-p300-CBP complex can bind to hypoxia-responsive elements (HREs) in the promoters of hypoxia-responsive target genes [13]. This process ultimately leads to the upregulation of a range of glycolytic enzymes, vascular endothelial growth factor (VEGF), erythropoietin (EPO), and other target genes [8, 12].

## 4. HIF-1α as a Regulator of Atrial Fibrillation

Acute AF is characterized by an estimated 2- to 3-fold increase in cardiomyocyte contractile and electrical activity, resulting in greater atrial oxygen and energy consumption [14]. Despite the significant increase in atrial blood flow relative to the sinus rhythm, cardiomyocytes experience relatively hypoxic conditions during AF episodes [15]. A clinical study revealed a significant increase in myocardial HIF-1α levels in the right auricle of patients with AF compared to those in sinus rhythm [16]. After comparing left atrial samples from patients with paroxysmal, persistent, and permanent atrial fibrillation to those with sinus rhythm, Xu *et al*. [17] found that the expression of HIF-1α in the left atrial tissues of patients with persistent or permanent atrial fibrillation was increased compared to those with paroxysmal atrial fibrillation or sinus rhythm. Together, these findings suggest that HIF-1α is readily expressed by cardiomyocytes under AF conditions, but also progressively upregulated as the disease advances.

### 4.1 HIF-1α Contributes to Atrial Electrical Remodeling

Electrical remodeling is central to the pathogenesis of AF, consisting primarily of changes in the expression and activity of a range of gap junction proteins and ion channels that result in abnormalities to cardiomyocyte repolarization, resting potential, excitability, and conductance [18]. The activity of HIF-1α is crucial for coordinating this electrical remodeling process. Sarcoplasmic reticulum Ca^2+^ adenosine triphosphatase (SERCA) is a major regulator of cardiomyocyte excitation-contraction coupling. In transgenic mice expressing an oxygen-stabilized isoform of HIF-1α, the cardiomyocytes exhibit marked reductions in SERCA 2a and ryanodine receptor 2 (RyR2) transcript levels [19]. These functional RyR2 defects can increase in the release of systolic sarcoplasmic reticulum-derived Ca^2+^ together with abnormally elevated cytosolic Ca^2+^ concentrations, leading to APD shortening and AF onset. While HIF-1α primarily exerts its functions by binding to HREs and inducing the transcription of target hypoxia-responsive genes, in some instances it can also suppress transcription in cases of reversed HRE orientation within a gene promoter [20].

HIF-1α can also reduce target gene expression via competitive inhibition mediated by HRE binding [21]. Ronkainen *et al*. [22] noted time-dependent reductions in cardiomyocyte SERCA 2a expression under hypoxic conditions (1% O_2_), and determined that desferrioxamine (DFO)-mediated HIF-1α activation or overexpression of a normoxia-stabilized heterodimeric form of HIF-1α (HIF-1α/VP16) was sufficient to suppress the endogenous expression and promoter activity of SERCA 2a. This aberrant SERCA 2a functionality, in turn, leads to higher cytosolic levels of Ca^2+^ and dysregulated Ca^2+^ activity (Ca^2+^ transients), culminating with subsequent disturbances such as delayed afterdepolarization (DAD) and AF [3]. Notably, the maintenance of SERCA activity necessitates the expenditure of approximately 15% of cardiac energy, suggesting that reductions in SERCA 2a expression may represent an adaptive response aimed at reducing energy expenditure during hypoxic conditions [22].

The Na^+^/Ca^2+^ exchanger 1 (NCX1), encoded by *Slc8a1*, is another key regulator of Ca^2+^ homeostasis [23]. Elevated levels of NCX1 protein have been observed in patients with AF, and an overly active NCX1 can promote action potential alternans, thereby increasing susceptibility to AF [23]. Wang *et al*. [24] found that FK506-binding protein 5 (FKBP5) is significantly under-expressed in atrial samples from patients with persistent long-term AF. They used *Fkbp5* knockout (*Fkbp5*^-⁣/-^) mice, which exhibited increased susceptibility to AF compared to controls. This is due to the fact that both FKBP5 and HIF-1α compete to bind with heat shock protein 90 (HSP90), and reduced FKBP5 expression increases the stability of HIF-1α. As a promoter of *Slc8a1*, HIF-1α upregulates NCX1 expression. Moreover, after treatment with an HSP90 inhibitor, the levels of HIF-1α and NCX1 proteins decreased in *Fkbp5*^-⁣/-^ mice. Most importantly, the rate of AF induction in *Fkbp5*^-⁣/-^ mice treated with the inhibitor was significantly lower compared to untreated *Fkbp5*^-⁣/-^ mice. These results suggest that elevated expression of HIF-1α, by enhancing its interaction with cardiac *Slc8a1*, promotes the occurrence of NCX1-mediated atrial arrhythmias.

### 4.2 HIF-1α Induces Atrial Structural Remodeling

Atrial fibrosis is a marker of atrial structural remodeling, characterized by the abnormal activation, proliferation, and differentiation of fibroblasts, as well as the excessive synthesis and irregular deposition of extracellular matrix proteins. Atrial fibrosis can be classified into two types: reactive fibrosis and reparative fibrosis.

Reactive fibrosis, a response to cardiac inflammation or pressure overload, manifests as perivascular and interstitial fibrosis [25]. It is commonly characterized by the activation of fibroblasts, which proliferate and differentiate into secretory myofibroblasts in response to various profibrotic stimuli. This process is typically accompanied by an upregulation of matrix metalloproteinases (MMPs) and a downregulation of tissue inhibitors of metalloproteinases (TIMPs). Ogi *et al*. [26] found that hypoxia-associated AF features upregulated HIF-1α and VEGF, which contributie to the enhanced expression of MMP-9. In a rabbit model of isoprenaline-induced AF, Su *et al*. [27] observed high levels of angiotensin-2, HIF-1α, transforming growth factor-β (TGF-β), and MMP-9 expression, while also noting a positive correlation between HIF-1α levels and the degree of myocardial fibrosis. Accordingly, the inhibition of HIF-1α expression resulted in corresponding decreases in TGF-β and MMP-9 expression, reducing the degree of myocardial fibrosis and thereby supporting the ability of HIF-1α to induce AF in part through the upregulation of MMP-9 and TGF-β. These abnormalities lead to an imbalance in the deposition and degradation of the extracellular matrix within the vascular space and cardiac interstitium, ultimately altering the ultrastructure of the heart.

Numerous studies have explored the mechanisms whereby HIF-1α can induce myocardial fibrosis in AF patients. Tsai *et al*. [28] noted that under hypoxic conditions, HIF-1α promotes AF by inducing phosphorylation of c-Jun N-terminal kinase (JNK) and activator of transcription factor 2 (ATF2), along with the concomitant upregulation of proteins associated with fibrosis. Chen *et al*. [29] demonstrated that HIF-1α can enhance miR-210 expression, inhibiting regulatory T cell (Treg) function via the targeting of FoxP3, contributing to AF. Furthermore, Abe *et al*. [30] noted that HIF-1α is capable of triggering inflammatory and fibrotic changes within epicardial adipose tissue by upregulating adipose angiopoietin-like protein 2 (ANGPTL2) expression, further contributing to AF progression. HIF-1α may thus function via multiple pathways to shape the atrial structural remodeling observed in AF.

Reparative fibrosis occurs after extensive loss of cardiomyocytes, and its role in the initiation and progression of atrial fibrillation remains controversial. Generally, scarring, primarily composed of fibroblasts and extracellular matrix, is generally considered to be non-conductive [31]. These collagen-based scars directly interfere with conduction, reducing the occurrence of atrial fibrillation [32]. However, at the infarct border zone, fibroblasts can couple with cardiomyocytes via connexin 43 (Cx43). Since fibroblasts have a lower membrane potential than the resting potential of the atria, they decrease the resting potential of the surrounding cardiomyocytes, thereby reducing the conduction velocity of action potentials and inducing AF. Among these mechanisms, HIF-1α may influence the expression of Cx43 and may thus facilitate the development and progression of AF [33].

### 4.3 HIF-1α Induces Atrial Fibrillation-Related Myocardial Metabolic Remodeling

In the absence of pathological changes, cardiomyocytes primarily rely on adenosine triphosphate (ATP) generated by mitochondrial oxidative phosphorylation as their main source of energy, with only a minor contribution from glycolysis [34]. Most glycolytic enzyme-encoding genes have been established as direct HIF-1α targets that can be induced under inflammatory or hypoxic conditions [35]. In hypoxic settings, HIF-1α is directly involved in the transitioning of cells between oxidative phosphorylation and glycolysis. Initially, HIF-1α can promote the upregulation of pyruvate dehydrogenase kinase 1 (PDK1), leading to the dephosphorylation of pyruvate dehydrogenase (PDH) involved in the tricarboxylic acid cycle and the blockade of pyruvate conversion into acetyl-coenzyme A (CoA). Furthermore, HIF-1α can induce the upregulation of glycolytic enzymes and the glucose transporter 1 (GLUT1) and glucose transporter 3 (GLUT3), enhancing glucose uptake within cells to help ensure an adequate supply of ATP [36].

HIF-1α can also drive lactate dehydrogenase A (LDHA) expression, resulting in the conversion of pyruvate to lactate and the regeneration of nicotinamide adenine dinucleotide (NAD)^+^ for further glycolytic cycling mediated by glyceraldehyde 3-phosphate dehydrogenase (GAPDH) [35]. Additionally, monocarboxylic acid transporter protein 4 (MCT4) plays a crucial role in the transport of lactate out of cells [37]. A shift away from oxidative phosphorylation in favor of glycolytic dependence results in a reduction in the consumption of oxygen necessary to produce ATP, leading to a drop in mitochondrial reactive oxygen species (ROS) biogenesis, shielding cells against oxidative injury [38, 39]. As glycolytic intermediates, lactate and pyruvate can also directly prevent the release of Ca^2+^ from the sarcoplasmic reticulum through a reduction in ryanodine receptor (RyR) activity [40]. Enhanced phosphofructokinase (PFK) activity can induce pathologic cardiac hypertrophy and influence the expression of key cardiac metabolism- and remodeling-related genes [41]. The enhanced production of lactate and associated lactate signaling activity have been established as a key regulator of atrial structural remodeling linked to oxidative stress-related damage and mitochondrial apoptosis [42].

Cardiac tissue primarily relies on fatty acid oxidation to generate 60–90% of its total ATP, with pyruvate oxidation contributing the remaining 10–40% [35]. In individuals with permanent AF, this metabolic balance is disrupted. Transcriptomic analyses have shown a downregulation of key enzymes involved in fatty acid oxidation [43]. Krishnan *et al*. [44] demonstrated that activation of the HIF1α-peroxisome proliferators-activated receptors γ (PPARγ) pathway leads to disrupted myocardial metabolism. This activation results in HIF-1α upregulating glycolytic genes, while PPARγ enhances glycolytic flux and the expression of fatty acid uptake genes, particularly affecting the glycerol-phosphate pathway. Concurrently, there is a decline in the expression of critical enzymes for fatty acid metabolism, such as carnitine palmitoyltransferase-1 (CPT-1), and a reduction in triglyceride oxidative utilization, leading to triglyceride accumulation and cardiac steatosis. Furthermore, HIF-1α can activate caspase-3 through the PPARγ/octamer-binding transcription factor 1 (Oct1)/growth arrest and DNA damage-inducible alpha (GADD45A) axis, triggering cardiomyocyte apoptosis. This cascade of events prompts compensatory responses, including myocardial hypertrophy and fibrosis, ultimately contributing to the development of AF.

## 5. Clinical Prospects and Challenges

Recent studies [16, 17] underscore the correlation between HIF-1α expression and AF incidence, suggesting that the HIF signaling axis may serve as a target for novel therapeutic interventions. Specifically, pharmacological modulation of HIF-1α activity can improve atrial structural and electrical remodeling, reducing the burden of AF. Metformin, for instance, is a commonly prescribed hypoglycemic drug that reportedly exerts cardioprotective activity. In an animal study, metformin administration improved cardiomyocyte lipid metabolism, a protective effect linked to the inhibition of HIF-1α expression and a subsequent reduction in downstream PPARγ levels mediated by activation of adenosine monophosphate activated protein kinase (AMPK) [45]. Furthermore, Bi *et al*. [46] determined that LDN-57444 can reduce LV remodeling, inflammation, and abrogating oxidative stress induced by angiotensin-2 by ubiquitin C-terminal hydrolase L1 (UCHL1). This ultimately curtailed AF incidence and duration by inhibiting the activation of atrial HIF-1α, TGF-β, and Smad 2/3 signaling. Several antitumor drugs have also been designed to target HIF-1α, including 32-134D, PX-478, and acriflavine. The safety and efficacy of these drugs in AF patients, however, has yet to be established [8, 47].

AF has a complex pathogenesis, and the specific contributions of HIF-1α warrant further study. While HIF-1α can reduce oxidative phosphorylation-mediated ROS biogenesis, under conditions of intermittent hypoxia it can also upregulate NADPH oxidase 2 (NOX2) and inhibit mitochondrial electron transport chain complexes I and III, resulting in higher levels of ROS production [48]. The degree to which HIF-1α can promote AF development, through changes in ion channel concentrations and autonomic nervous function, will also require further study. Moreover, comprehensive efforts are needed to characterize the links between common therapeutic agents, including metformin or antiarrhythmic drugs, and HIF-1α. These studies will be critical for the production of new drugs that can aid in the prevention and treatment of AF.

## 6. Conclusions

**Fig. 1.**
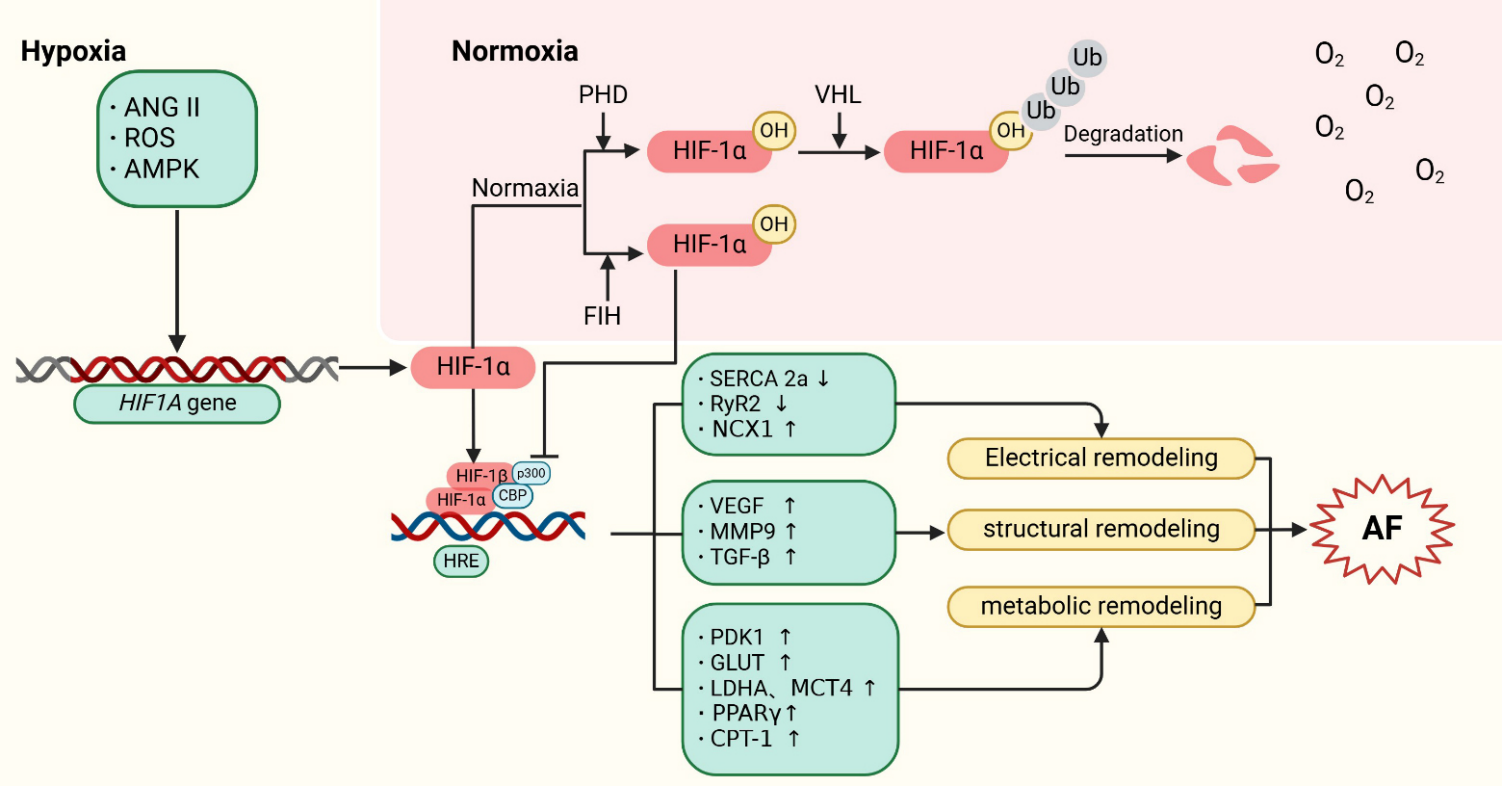
**Mechanisms of HIF-1α regulation in atrial fibrillation**. HIF-1α is encoded by the *HIF1A* gene. Under normoxic conditions, HIF-1α is hydroxylated by PHDs, leading to its subsequent proteasomal degradation following ubiquitination by pVHL. Additionally, factor inhibiting HIF (FIH) hydroxylates an asparagine residue on HIF-1α, reducing its interaction with CBP/p300 and thus inhibiting its transcriptional activity. Under hypoxic conditions, upstream factors such as ANGII, ROS, and AMPK stimulate the expression of the *HIF1A* gene. Concurrently, the activities of PHDs and FIH are reduced, allowing HIF-1α to translocate to the nucleus. There, it forms a complex with HIF-1β and CBP/p300, and binds to hypoxia-response elements (HREs) in the promoter regions of HIF target genes. This interaction activates the expression of downstream proteins, contributing to the development of AF. Specifically, HIF-1α promotes atrial structural remodeling by inducing the expression of target genes such as VEGF, MMP9, and TGF-β. It also drives atrial metabolic remodeling through the upregulation of PDK1, GLUT, LDHA, MCT4, PPARγ, and CPT-1. Furthermore, HIF-1α’s binding to HRE sequences competitively inhibits the expression of SERCA 2a and RyR2, thereby promoting atrial electrical remodeling. ANGII, angiotensin II; ROS, reactive oxygen species; AMPK, adenosine monophosphate activated protein kinase; HIF, hypoxia-inducible factor; PHD, prolyl-4-hydroxylases; pVHL, von hippel-lindau proteins; HRE, hypoxia response elements; FIH, factor inhibiting HIF; SERCA, sarcoplasmic reticulum Ca^2+^ ATPase; RyR2, ryanodine receptor 2; NCX1, Na^+^/Ca^2+^-exchanger 1; VEGF, vascular endothelial growth factor; MMP9, matrix metalloproteinase 9; TGF-β, transforming growth factor β; PDK1, pyruvate dehydrogenase kinase 1; GLUT, glucose transporters; LDHA, lactate dehydrogenase A; MCT4, monocarboxylate transporter 4; PPARγ, peroxisome proliferators-activated receptors γ; CPT-1, carnitine palmitoyl transferase-1; AF, atrial fibrillation; VHL, von hippel-lindau; Ub, ubiquitin; CBP, CREB-bindingprotein.

In conclusion, HIF-1α is an essential regulator in AF pathophysiology. At the cellular level, HIF-1α can contribute to the exacerbation of atrial structural, electrical, and metabolic remodeling which disrupt normal electrophysiological activities, cellular structures, and cardiomyocyte energy metabolism. These adverse modifications perpetuate a deleterious feedforward cycle and a worsening of AF (Fig. 1). Interventional strategies focused on targeting HIF-1α hold promise as a means of managing patients suffering from this form of arrhythmia. The modulation of HIF-1α activity may help disrupt the progressive electrophysiological and metabolic deterioration that characterizes AF progression within the atria, thereby preventing disease progression and potentially reversing the course of the disease. However, further research is essential to determine the safety and efficacy of these treatments, aiming to provide AF patients with more efficacious and precise pharmacological options for disease management.
